# Evaluation of expiratory capacity with severe asthma following bronchial thermoplasty

**DOI:** 10.1002/rcr2.387

**Published:** 2018-11-26

**Authors:** Satoru Ishii, Motoyasu Iikura, Yukiko Shimoda, Shinyu Izumi, Masayuki Hojo, Haruhito Sugiyama

**Affiliations:** ^1^ Department of Respiratory Medicine National Center for Global Health and Medicine Tokyo Japan; ^2^ Division of Respirology NTT Medical Center Tokyo Tokyo Japan

**Keywords:** Expiratory capacity, bronchial thermoplasty, severe asthma

## Abstract

Bronchial thermoplasty (BT) is a bronchoscopic treatment to reduce the amount of smooth muscle in the bronchial wall in patients with severe asthma. Reducing smooth muscle in the airway wall reportedly alleviates air trapping and decreases expiratory volume. In the current study, expiratory computed tomography (CT) was performed in 10 patients who underwent BT at our facility, and their expiratory volume was evaluated. We observed an improvement in the expiratory volume on CT in nine of the 10 patients. Total expiratory lung volume decreased from 1693 ± 907 to 1426 ± 853 mL, indicating an improvement of approximately 15%. Use of CT for evaluation of expiratory volume may be a method for assessing the effectiveness of BT.

## Introduction

Bronchial thermoplasty (BT) is a bronchoscopic treatment for severe asthma using thermal energy to reduce the amount of smooth muscle in the bronchial wall. Many studies have found that it improves symptoms in patients with severe asthma 1, 2, 3. Although severe asthma is a disease that causes obstructive ventilatory impairment through thickening of central airway walls, very few reports have compared expiratory lung capacity before and after BT. In the present study, expiratory computed tomography (CT) imaging was performed before and after BT, and changes in expiratory lung capacity were measured.

## Case Series

### Methods

Assessments including respiratory function tests and CT were performed approximately 2 weeks before the first session of BT and again approximately 4 weeks after the third session of BT. This work was performed with the approval of the Ethics Committee of the National Center for Global Health and Medicine (NCGM‐G‐001801‐00). Written informed consent was obtained from each participant. An Olympus P240 or type260 bronchofiberscope (Olympus, Tokyo, Japan) was used with an Alair Bronchial Thermoplasty System (Boston Scientific Corporation, Tokyo, Japan) as the high‐frequency energization system. CT was carried out with an Aquilion ONE™ TSX‐301A (Toshiba Medical Systems, Tokyo, Japan). The CT threshold was set at −960 HU. Expiratory CT was taken by maximum exhalation level. Expiratory lung capacity was analysed using Virtual Place (AZE, Tokyo, Japan). Virtual Place is equipped with the capability to automatically measure expiratory lung capacity. Pulmonary function tests (forced vital capacity (FVC), forced expiratory volume in 1 second (FEV1), and others) were carried out using computerized equipment (model CHESTAC‐8100; CHEST MI, Inc., Tokyo, Japan).

### Results

**Figure 1:**
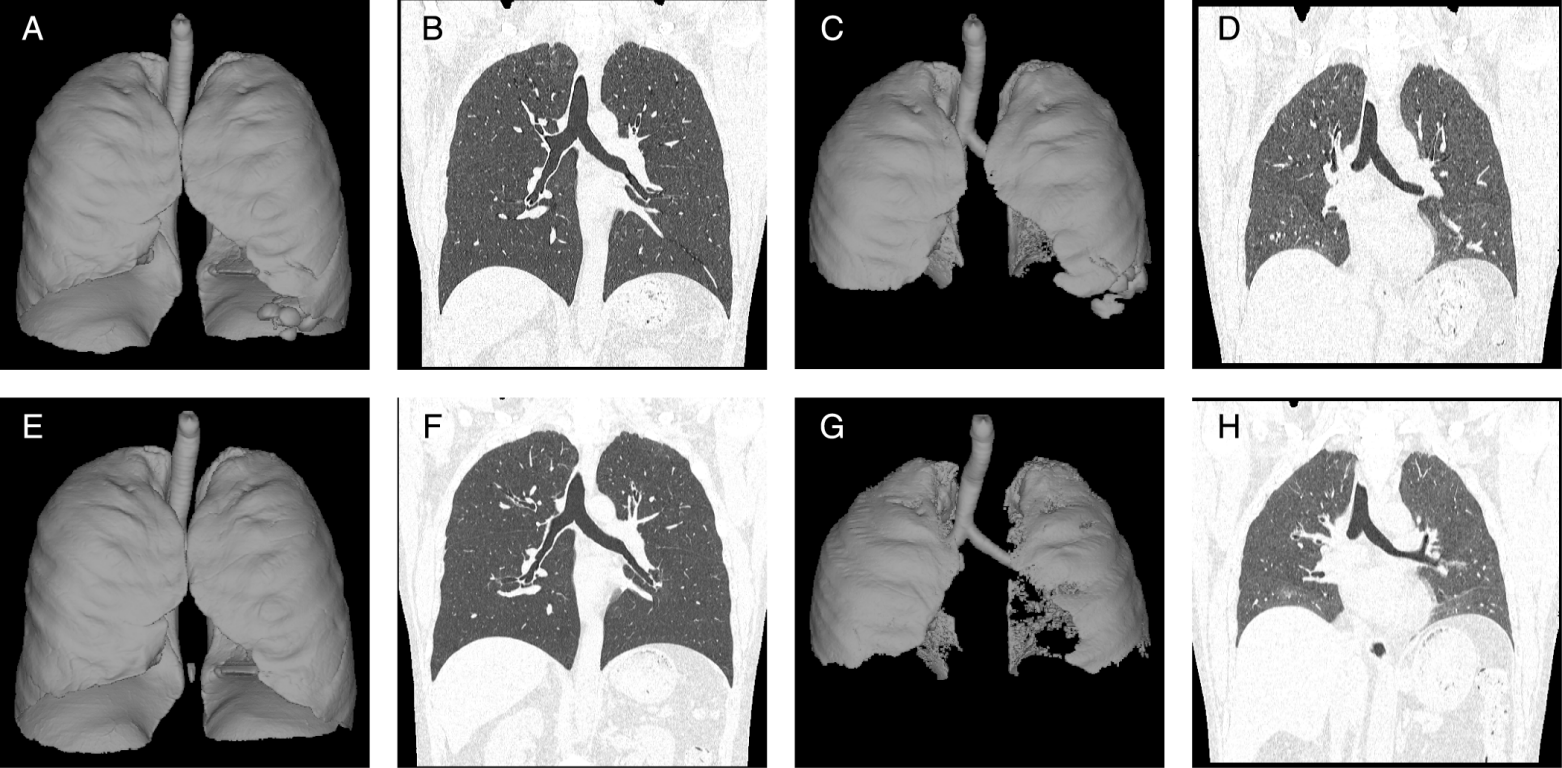
Lung capacity by Virtual Place in the inspiration and expiration. Case: A 44‐year‐old man; asthma disease duration: eight years; lung total volume on inspiration before bronchial thermoplasty (BT): 5873 mL. (A) Computed tomography (CT) coronal slices on inspiration before BT; (B) lung total volume on expiration before BT 2226 mL; (C) CT coronal slices on expiration before BT; (D) lung total volume on inspiration after BT 5660 mL; (E) CT coronal slices on inspiration after BT; (F) lung total volume on expiration after BT 1067 mL; (G) CT coronal slices on expiration after BT (H).

All subjects met the European Respiratory Society (ERS)/American Thoracic Society (ATS) definition of severe asthma and required regular maintenance medications using high‐dose inhaled corticosteroid (ICS) and long‐acting beta‐2 agonists (LABA), according to Global Initiative for Asthma (GINA) treatment step 4 or 5. Consent was obtained from 10 patients (six men, four women; mean age 55.5 (range: 26–77); body mass index 25.2 (range: 18–34)). Two patients had a smoking history, eight patients did not. The mean duration of asthma was 31 (range: 8–63). Four patients were receiving GINA step 4 treatment, and six patients were receiving GINA step 5 treatment. Systemic steroid therapy was used in 40% of the patients, and omalizumab was used (including past use) in 70% of the patients. ICS, LABA, or leukotriene receptor antagonist (LTRA) treatment was given to 100% of the patients, with theophylline in 90% and long‐acting muscarinic receptor antagonist (LAMA) in 70%. Approximately 4 weeks after the third session of BT, FVC (l) did not change from 3.0 ± 0.9 to 3.1 ± 0.9, FEV_1_ (%predicted) increased mildly from 84.4 ± 21.9 to 87.9 ± 19.4, functional residual capacity (FRC) (%predicted) decreased mildly from 87.2 ± 18.1 to 83.6 ± 18.0, and residual volume (RV) (%predicted) decreased mildly from 104 ± 18.5 to 97.5 ± 14.6 (Table 1). We observed an improvement in the expiratory volume on CT in nine of the 10 patients. Total expiratory lung volume (mL) decreased from 1693 ± 907 to 1426 ± 853, an improvement of approximately 15%. See the example of one patient illustrated in Figure 1. The Asthma Quality of Life Questionnaire (AQLQ) score improved from 4.0 ± 1.5 to 5.2 ± 1.8. Statistics are not performed at all date, because the number of cases is small and statistics are impossible.

**Table 1:** Patient characteristics and effectiveness outcomes.

Characteristics of subjects (*n* = 10)	
Age (years)	55.5 ± 18.6
Sex, *n* (%)	
Men	6 (60)
Women	4 (40)
Height (cm)	158.6 ± 7.0
Body weight (kg)	63.5 ± 13.1
BMI	25.2 ± 5.4
Smoking status, *n* (%)	
Never‐smoker	8 (80)
Ex‐smoker	2 (20)
IgE (IU/mL)	201.5 ± 276.8
Disease duration (years)	31.1 ± 17.6
GINA treatment step, *n* (%)	
4	4 (40)
5	6 (60)
Systemic steroid use, *n* (%)	4 (40)
Omalizumab, *n* (%)	used 3 (30); not used 3 (30); used it previously 4 (40)
ICS use, *n* (%)	10 (100)
LABA use, *n* (%)	10 (100)
LAMA use, *n* (%)	7 (70)
LTRA use, *n* (%)	10 (100)
Theophylline use, *n* (%)	9 (90)

	Before BT	After BT
FVC (l)	3.0 ± 0.9	3.1 ± 0.9
FEV1 (%predicted)	84.4 ± 21.9	87.9 ± 19.4
RV (%predicted)	104.2 ± 18.5	97.5 ± 14.6
FRC (%predicted)	87.2 ± 17.2	83.6 ± 18.0
Total lung volume on expiratory (mL)	1693 ± 907	1426 ± 853
MLD	−815 ± 31.3	−816 ± 30.9
AQLQ score	4.0 ± 1.5	5.2 ± 1.8

Values are presented as mean ± SD.

AQLQ, Asthma Quality of Life Questionnaire; FRC, functional residual capacity; LAMA, long‐acting muscarinic receptor antagonist; LTRA, leukotriene receptor antagonist; MLD, mean lung density; RV, residual volume.

## Discussion

These data demonstrate, for the first time, the usefulness of expiratory CT imaging by Virtual Place performed before and after completing three sessions of BT, and the changes in expiratory lung capacity measured in Japan. Generally, indices such as the number of asthma exacerbations and AQLQ score are used to assess the effectiveness of BT. Although FEV_1_ is also used for this, the Asthma Intervantion Research 2 (AIR2) trial, which excluded patients with an FEV_1_ <60% of the predicted value, reported that FEV_1_ remained unchanged for 5 years after BT 4. On the other hand, another study found that BT improved FEV_1_ in a group that also included patients with an FEV_1_ ≤60% 1. Therefore, since the results vary depending on the cut‐off FEV_1_ value that is used, FEV_1_ is not a suitable index for assessing the effectiveness of BT. Pretolani et al. biopsied the bronchial wall before and after BT, and reported a decrease in airway smooth muscle after BT 5. Virtual Place can measure the airway wall, as well as lung capacity, as shown in our previous report 6. Reduction of airway smooth muscle alleviates air trapping, decreasing expiratory volume. Examination of the changes in expiratory volume may thus be an index with which to assess the effectiveness of BT. Several reports have demonstrated the usefulness of evaluating expiratory CT in addition to inspiratory CT. Ueda et al. suggested that the evaluation of air trapping using high‐resolution expiratory and inspiratory CT may be useful in the early diagnosis of asthma 7. Zanon et al. evaluated expiratory CT using the software Advantage Workstation 4.6, GE Healthcare in 26 patients before and one year after BT, and they reported that lung capacity improved from 2668 to 2399 mL 8. In our study, total expiratory lung volume decreased from 1693 ± 908 to 1427 ± 853 mL after completion of three sessions of BT, showing improvement in air trapping and increased ability to exhale, although the measurements were made 4 weeks after the third BT session. These findings suggest that improvement of expiratory volume continues from after the completion of three sessions of BT to one year after. Although CT evaluations are useful, they involve radiation exposure. Thomen et al. reported that regional lung ventilation after BT can be evaluated using helium‐3 magnetic resonance 9. In future, long‐term follow‐up for at least one year in addition to short‐term follow‐up is necessary to confirm the benefits of BT. Determination of the most useful type of imaging evaluation is also necessary in future studies.

There are several limitations to the present study. First, the sample size was small. Second, the patients were taking high‐dose oral steroids as pre‐treatment for BT, which may have affected the results. Third, evaluation was performed one month after the third BT in the present study; since this was soon after the third BT, inflammation associated with BT might still have been present. This could be the reason why changes in mean lung density (MLD) were not noted. It is therefore unclear whether an evaluation at this time point is appropriate in all cases.

In conclusion, this study indicated the utility of expiratory CT evaluation in 10 patients with persistent asthma who underwent BT. Use of CT to evaluate expiratory volume may be a way to assess the effectiveness of BT.

## Disclosure Statement

This work was performed with the approval of the Ethics Committee of the National Center for Global Health and Medicine (NCGM‐G‐001801‐00), and was designed in accordance with the Helsinki declaration. M. Iikura received lecture fees from Boston Scientific Japan. The rest of the authors have no conflict of interest.
